# Peptidoglycan Recognition Protein 4 Limits Bacterial Clearance and Inflammation in Lungs by Control of the Gut Microbiota

**DOI:** 10.3389/fimmu.2019.02106

**Published:** 2019-09-20

**Authors:** Alexander N. Dabrowski, Anshu Shrivastav, Claudia Conrad, Kassandra Komma, Markus Weigel, Kristina Dietert, Achim D. Gruber, Wilhelm Bertrams, Jochen Wilhelm, Bernd Schmeck, Katrin Reppe, Philippe D. N'Guessan, Sahar Aly, Norbert Suttorp, Torsten Hain, Janine Zahlten

**Affiliations:** ^1^Department of Infectious Diseases and Respiratory Medicine, Charité – Universitätsmedizin Berlin, Corporate Member of Freie Universität Berlin, Humboldt-Universität zu Berlin, Berlin Institute of Health, Berlin, Germany; ^2^Institute of Medical Microbiology, Justus-Liebig University Giessen, Giessen, Germany; ^3^Department of Veterinary Pathology, Freie Universität Berlin, Berlin, Germany; ^4^Institute for Lung Research/iLung, Universities of Giessen and Marburg Lung Center, Member of the German Center for Lung Research, Philipps University Marburg, Marburg, Germany; ^5^Excellence Cluster Cardio Pulmonary System, The German Center for Lung Research, Justus-Liebig University Giessen, Giessen, Germany; ^6^Division of Pulmonary Inflammation, Charité – Universitätsmedizin Berlin, Corporate Member of Freie Universität Berlin, Humboldt-Universität zu Berlin, and Berlin Institute of Health, Berlin, Germany; ^7^German Centre for Infection Research (DZIF), Partner Site Giessen-Marburg-Langen, Giessen, Germany

**Keywords:** infectious diseases, innate immunity, microbiome, peptidoglycan recognition proteins (PGRP, PGLYRP), pneumococcal pneumonia, *Streptococcus pneumoniae*

## Abstract

*Streptococcus pneumoniae* is the most frequent cause of community-acquired pneumonia. Endogenous host defense molecules such as peptidoglycan recognition protein 4 (PGLYRP4) might influence the course of this disease. To the best of our knowledge, there are no reports on the relevance of PGLYRP4 in pneumonia. Therefore, wild type (WT) and PGLYRP4-deficient (PGLYRP4KO) mice were analyzed in an *in vivo* and *in vitro* experimental setting to examine the influence of PGLYRP4 on the course of pneumococcal pneumonia. Furthermore, caecal 16S rRNA microbiome analysis was performed, and microbiota were transferred to germfree WT mice to assess the influence of microbiotal communities on the bacterial burden. Mice lacking PGLYRP4 displayed an enhanced bacterial clearance in the lungs, and fewer mice developed bacteremia. In addition, an increased recruitment of immune cells to the site of infection, and an enhanced bacterial killing by stronger activation of phagocytes could be shown. This may depend partly on the detected higher expression of complement factors, interferon-associated genes, and the higher pro-inflammatory cytokine response in isolated primary PGLYRP4KO vs. WT cells. This phenotype is underlined by changes in the complexity and composition of the caecal microbiota of PGLYRP4KO compared to WT mice. Strikingly, we provided evidence, by cohousing and stable transfer of the respective WT or PGLYRP4KO mice microbiota into germfree WT mice, that the changes of the microbiota are responsible for the improved clearance of *S. pneumoniae* lung infection. In conclusion, the deficiency of PGLYRP4, a known antibacterial protein, leads to changes in the gut microbiota. Thus, alterations in the microbiota can change the susceptibility to *S. pneumoniae* lung infection independently of the host genotype.

## Introduction

*Streptococcus pneumoniae* (*S. pneumoniae*) was discovered in the late nineteenth century (1) as a major course of pneumonia. It kills 1.5–2 million people every year with the highest rates in children younger than five and adults older than 65 years of age (2, 3). Thus, *S. pneumoniae* is one of the major causes of death worldwide. The global increase of antibiotic resistant *S. pneumoniae* strains (4), and escape strategies to vaccination via serotype-switch (5) underline the necessity to develop new treatment strategies.

Host defense molecules (HDMs) such as defensins, cathelicidine LL-37, and peptidoglycan recognition proteins (PGRPs/PGLYRPs) were shown to have not only direct antimicrobial activity, but often also act in an immunomodulatory fashion (6). PGRPs were first isolated in 1996 from the silkworm *Bombyx mori* (7) and mice (8). Now, about a hundred PGRPs have been identified (9, 10). For the purpose of distinction, mammalian PGRPs have been termed as PGLYRPs and numbered from 1 to 4. They have either an *N-*acetylmuramyl-alanine amidase activity (PGLYRP2), or are described to have antibacterial properties (PGLYRP1-4) against a range of gram-positive and gram-negative bacteria (10–14).

Furthermore, several groups reported immunomodulatory functions including chemoattractant and cytotoxic properties, antitumor effects, regulation of pro-inflammatory cytokine response in chemical-induced inflammation models, and influence on the recruitment of PMNs (15–20). PGLYRP4 especially is known for its regulation on the Treg/Th17 balance and anti-inflammatory properties in atopic dermatitis and chemical-induced colitis (18). PGLYRP4 is mainly expressed in epithelial cells of the esophagus, skin, and intestine (10). Furthermore, there are also reports of a regulatory role of PGLYRP4 on healthy gut microbiota (19, 21).

The microbiota is considered to include all microbes (bacteria, archaea, viruses, fungi, and protozoa) associated with and within the organism (22). Interaction between host and the microbiota is an important factor for the host's health. Shifts or imbalances of the microbiota, also called dysbiosis, can be followed by pathogenic bacteria outgrowing the commensals, and ultimately promoting or even causing diseases (23, 24).

Increasing number of reports highlight the influence of the gut microbiota on lung diseases. Thus, the so-called gut-lung axis has become an important topic in microbiota research in the last few decades. Current research indicates an important role of the gut microbiota in the defense against lung infections such as bacterial pneumonia (25), or have linked chronic obstructive pulmonary disease (COPD) to Crohn's disease (26). Such effects could be mediated, at least partly, by bacterial metabolites like short-chain fatty acids (SCFAs), which can have systemic immunomodulatory effects by migration via the blood stream (27).

To analyze the so far unknown effects of PGLYRP4 on lung infections, and investigate possible gut-lung immune interactions, we investigated the influence of PGLYRP4-deficiency on the development and outcome of pneumococcal pneumonia. Therefore, we transnasally infected WT and PGLYRP4KO mice with *S. pneumoniae*. Unexpectedly, we found a lower bacterial burden in the lungs and blood of infected PGLYRP4KO vs. WT mice, which was accompanied by higher inflammation, and enhanced bacterial killing by phagocytes. Cohousing experiments confirmed that these effects are at least partly mediated by the microbiota.

## Materials and Methods

### Animals

#### Source of Animals

All mice were on a BALB/c background, bred and housed at the central breeding facility of the Charité – Universitätsmedizin Berlin (Forschungseinrichtung für Experimentelle Medizin, FEM). Breeding pairs for the WT and PGLYRP4KO mice were kindly provided by Prof. Dr. Roman Dziarski (Department of Microbiology and Immunology, Indiana University School of Medicine, Indiana, USA) who originally obtained the mice from Harlan-Sprague-Dawley and generated the PGLYRP4KO mice as described by Saha et al. (19). Germ-free BALB/c WT mice were obtained from the central animal facility of the Medical University of Hannover.

#### Housing Conditions

Animals were kept at a 12 h light/dark cycle, with food and water *ad libitum*. All animal procedures were in compliance with the Federation of European Laboratory Animal Science Associations (FELASA) guidelines and recommendations for the care and use of laboratory animals, and were approved by local institutional (Charité – Universitätsmedizin Berlin) and governmental (LAGeSo Berlin, approval ID: G0251/12 and G0266/15) authorities.

PGLYRP4KO mice were routinely genotyped to ensure that they were homozygous for the knockout. We did not use heterozygous WT and KO littermates because the effect of the PGLYRPs on the composition of the microbiota takes a long time to stabilize, which is only possible with homozygous breeding [described earlier in Dziarski et al. (21)].

It is known that housing conditions such as diet, stress, type of caging, and other environmental factors have an impact on the gut microbiome (28, 29). Therefore, PGLYRP4KO and WT mice were bred and housed in the same conventional pathogen-free room of our facility with the same environment setting to avoid differences in the microbiota by variations in the housing conditions. Individually ventilated cages, nesting material, enrichment, food and water were autoclaved. Furthermore, all experiments were done with animals from several breeding pairs to avoid analyzing selective microbiota from only one maternal lineage or cage and to increase biological plausibility by increased diversity.

#### Additional Information

**Figure 1:**
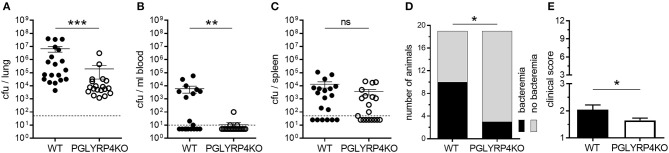
Lower bacterial burden in lungs and blood of PGLYRP4KO mice. Mice were infected with *S. pneumoniae* (NCTC 7978, 10^5^ cfu) for 48 h and **(A)** the lungs, **(B)** the blood, and **(C)** the spleens were analyzed for the bacterial load. In addition, **(D)** the number of bacteremic mice and **(E)** the clinical score (min.: 1, max.: 12) was assessed. A total of 19 mice were used in **(A–D)** and 23 mice (including 4 mice from histological analysis) in **(E)**. In **(A–C)**, the bars represent the means ± SEMs and in **(E)** the mean + SEM is given. The dotted lines represent the lower limit of detection (LLOD). Undetected samples were set to half of the LLOD for statistical purposes. Statistical analysis: **(A–C)** and **(E)**: Student's *t*-test on logarithmic data and **(D)** Chi^2^-test; ^*^p ≤ 0.05, ^**^p ≤ 0.01, ^***^p ≤ 0.001, ^ns^*p* ≥ 0.1.

**Figure 2:**
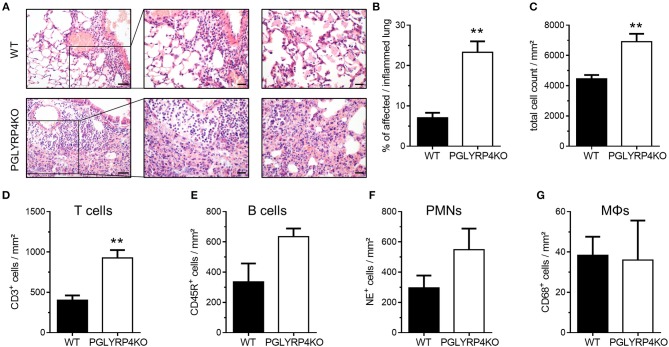
Histopathological and digital image analyses of infected WT and PGLYRP4KO mouse lungs. The lungs of infected (*S. pneumoniae*, NCTC 7978, 10^5^ cfu, 48 h post infection) mice were harvested, fixed in formalin, and embedded in paraffin. Afterwards, 2 μm sections were prepared and stained with **(A–C)** hematoxylin and eosin for histopathological analyses or **(D–G)** CD68, NE, CD45R, or CD3 antibodies for quantification of immune cell populations in the lung. **(A)** Representative images for WT and PGLYRP4KO are shown (*n* = 3–4 per group). Bar (left panel), 100 μm. Bar (center and right panel), 50 μm. The panels are showing representative areas of the perivascular area (left and center) and the alveolar compartment (right panel). Quantitative digital image analysis on digitalized whole lung sections was performed by using an adapted GENIE pattern recognition algorithm and v9 nuclear count algorithm and **(B)** the percentages of affected/inflamed lung areas and **(C)** total cell counts in the lungs were assessed. Further, total cell count per lung area of **(D)** CD68^+^ (macrophages/monocytes), **(E)** NE^+^ (neutrophils), **(F)** CD45R^+^ (B cells), and **(G)** CD3^+^ (T cells) were digitally quantified. Values are expressed as means + SEMs. Statistical analysis: Student's *t*-test: ^**^p ≤ 0.01 vs. infected WT.

PGLYRP4KO and respective control WT mice were always infected together to exclude differences due to infection time, dose, or bacteria. In accordance to the 3R principles we reduced the amount of animals by using some of the WT mice also in another study with PGLYRP3-deficent mice (30). These include 11 WT animals in Figures 1A–D, 7A–C,E, 15 WT mice in Figures 1E, 7D, and three WT mice in Figure 2A. Thereby, we reduced the number of animals used in these studies to the minimum amount.

### Genotyping PCR

Tail tips were used for the isolation of DNA. After incubation over night at 54°C in tail lysis buffer (0.1 M Tris, 5 mM EDTA, 200 mM NaCl, 0.2% SDS, 10 mg/ml Proteinase K), the samples were then centrifuged (4,000 × g, 10 min, RT). The DNA was extracted from the supernatant with isopropanol, washed twice with 70% ethanol, air dried, and dissolved in nuclease-free water (56°C, 30 min).

The PCR was performed using 50 ng of purified DNA, 0.1 mM dNTPs, 0.1 μM of each forward and reverse primer, and 1.25 U DreamTaq DNA Polymerase. Primers were as follows: Forward primer either WT: GCACTCTAGTCCAGGGATATGGTG or PGLYRP4KO: AGCGCATCGCCTTCTATCGCCTTC and reverse primer GTCAGTGTCTCTCCTAGGGTTGC (TIB MOLBIOL GmbH, Berlin, Germany). The cycling conditions were: 3 min at 95°C, 30 cycles with 30 s at 95°C, 30 s at 64°C and 2 min at 72°C, plus a final step for 7 min at 72°C. After amplification, PCR products were run on a 1% agarose gel (Promega, Mannheim, Germany) with 0.04% ethidium bromide (Invitrogen, Massachusetts, USA), and visualized under UV-light to detect the amplicons.

### Mouse Infection

#### Infection Model

For the *in vivo* experiments, female BALB/c mice (9–12 weeks) were used. After anesthesia with i.p. injection of ketamine and xylazine (both Rotexmedica, Luitré, France), mice were inoculated transnasally with 20 μl of bacterial suspension of *S. pneumoniae* serotype three strain (NCTC 7978) or 20 μl of sterile PBS as a control (30).

#### Monitoring

During the experiments, the mice were monitored every 12 h to assess clinical signs of illness Humane endpoints were defined as (i) body weight loss ≥ 20%, (ii) cumbersome breathing, and (iii) accelerated breathing or hypothermia (<30°C) in combination with staggering or pain. No mice reached the predefined humane endpoints before the end of the experiments.

#### Scoring of Clinical Signs

The following 11 symptoms were scored: disheveled fur, crusty secretion on the eye rim, reduced reaction or movement, isolation, temperature ≤ 35.0°C, body weight loss ≥ 10%, body weight loss ≥ 20%, body weight loss ≥ 25%, accelerated breathing, cumbersome breathing, pain. Scoring was performed according to their presence or not (1 or 0, respectively) with the exemption of disheveled fur, which was scored according to its severity (0, 0.5, 1, 1.5, or 2) and the sum was calculated. For statistical purpose, 1 was added to the sum of the scores to result in a final score ranging from 1 (minimum) to 12 (maximum).

### Bacterial Strains

NCTC 7978 (A66) and NCTC 7466 (D39) were plated on Columbia agar plates with 5% sheep blood and incubated overnight (37°C, 5% CO_2_). Single colonies were inoculated in Todd-Hewitt broth, 0.5% yeast extract (both BD Biosciences, Heidelberg, Germany), and 10% heat-inactivated FCS. After incubation (37°C, 5% CO_2_) until the mid-logarithmic growth phase, the bacteria were centrifuged (1,800 × g, 10 min) and the bacterial pellet was resuspended in cell culture medium (*in vitro* stimulations) or in sterile PBS (*in vivo* infections) in appropriate concentration (30).

### Bacterial Load

Serial dilutions of lung and spleen homogenates or EDTA-blood from the *in vivo* experiments were plated on Columbia agar with 5% sheep blood and incubated overnight (37°C, 5% CO_2_)_._ Colonies were counted manually, and the bacterial load as colony forming units (cfu) was calculated.

### Cohousing Experiment

For microbiota transfer, 3–4-week-old female WT or PGLYRP4KO mice were cohoused with female germ-free WT mice at a ratio 1:1 with 2–6 mice per cage until an age of 9-10 weeks. Cages were changed weekly under an UV irradiated laminar flow bench, and all materials including food and water were autoclaved for sterility.

### Isolation of Primary Cells

Untreated BALB/c WT and PGLYRP4KO mice (male, age 10–16 weeks) were anesthetized i.p. with ketamine and xylazine and exsanguinated by opening the *vena cava caudalis*. For the isolation of AECs heparin (Rotexmedica, Luitré, France) was included in the anesthesia mix.

#### Isolation of Alveolar Epithelial Cells (AECs)

The lungs were perfused via the heart with PBS, instilled with 5,000 U of Dispase (BD Biosciences, Heidelberg, Germany), and low melt agar (Invitrogen, Massachusetts, USA) as described previously with some modifications (31). After incubation in Dispase (6 min, 37°C), the lungs were macerated and homogenized by passing through cell strainers (100, 70, and 30 μm). The cell suspension was centrifuged (200 × g, 10 min, 4°C) and resuspended in PBS (3% FCS, 10 mM EDTA).

The AECs were isolated via negative selection with the following antibodies: CD45, CD31, CD16/32, all biotinylated (BD Biosciences, Heidelberg, Germany) using the MACS procedure according to manufacturer's instructions (Miltenyi Biotec GmbH; Telterow, Germany). The purified AECs were centrifuged (200 × g, 10 min, 4°C), resuspended in DMEM (1% P/S, 1% Glu, 25 mM HEPES, 10% FCS), seeded into cell culture plates, and incubated (37°C, 5% CO_2_, overnight). The next day, medium was changed to DMEM with 2% FCS and 1% Glu and the cells were infected 2 h later.

#### Isolation of Alveolar Macrophages (AMΦs)

Alveolar macrophages were isolated from bronchoalveolar lavage fluid as described (31). The AMΦs were incubated (37°C, 2 h) in cell culture plates and medium (RPMI 1640, 10% FCS, 1% P/S, 1% Glu) was changed to remove non-adherent cells. After overnight incubation (37°C, 5% CO_2_) the medium was changed to RPMI 1640 with 2% FCS and 1% Glu and the cells were infected 2 h later.

#### Isolation of Neutrophils (PMNs)

Cells were flushed out from the femurs and tibiae with sterile PBS, and PMNs were isolated via positive selection using the MACS mouse Anti-Ly-6G Microbead kit (Miltenyi Biotech, Bergisch Gladbach, Germany) according to the manufacturer's instructions. The isolated neutrophils were resuspended in RPMI 1640 with 2% FCS, 1% Glu and stimulated immediately.

### Quantitative Real-Time PCR (qPCR)

For RNA isolation from primary BALB/c WT, AECs, AMΦs, and PMNs the TRIzol method was used. Total RNA (1 μg) was transcribed into cDNA (High-Capacity cDNA Reverse Transcription Kit), pre-amplified (TaqMan PreAmp Master Mix Kit), and used for qPCR (TaqMan Gene Expression Master Mix and TaqMan Assay Sequence Numbers: *Gapdh* Mm99999915_g1, *Pglyrp4* Mm01220032_m1, *Cldn18* Mm00517321_m1, *F11r* Mm00554113_m1, *Cdh1* Mm01247357_m1, *C3* Mm01232779_m1, *Ifng* Mm01168134_m1, *Ifngr*1 Mm00599890_m1; all Life Technologies GmbH, Darmstadt, Germany). The qPCR was performed according to the manufacturer's instructions. Cycling conditions: 2 min, 50°C, 10 min, 95°C, and 40 cycles with 15 s, 95°C and 1 min, 60°C. Relative expression was calculated by the efficiency corrected ΔΔCt method (32) with *Gapdh* as a housekeeping gene, and uninfected WT cells as an untreated control.

### Cytokine Measurement

Cell-free supernatants were used to measure the cytokines from cultured primary WT and PGLRP4KO mouse cells (AECs, AMΦs, and PMNs). All ELISAs were performed according to the manufacturer's instructions (KC from R&D Systems, Abingdon, UK; all others from eBioscience, Frankfurt am Main, Germany). Values were adjusted to the mean of infected WT cells.

### Microarray

Primary WT and PGLYRP4KO AMΦs and AECs were cultured, infected, and RNA was extracted as described above. For the AMΦs, RNA of three independent experiments were sent to Source BioScience imaGene (Berlin, Germany) for analysis on an Affimetrix GeneChip Mouse Gene 2.0 ST Array. For AECs, three independent experiments were pooled. Analysis was performed as described (33, 34) on an Agilent SurePrint G3 ^*^ Mouse ^*^ Gene Expression 8x60K v2 Microarray with 200 ng RNA per sample.

### Histology of the Lung

Mice were anesthetized i.p. with ketamine and xylazine, and heparinized before exsanguination. Whole lungs including tracheas were removed after ligation of the trachea to prevent alveolar collapse, and immediately immersion-fixed in 4% buffered formalin (Sigma-Aldrich, Darmstadt, Germany) at pH 7 for up to 48 h. Afterwards, lungs were embedded in paraffin and cut into 2 μm sections.

For histopathology and immunohistochemistry (IHC) for the identification of immune cell populations, sections were dewaxed in xylene, rehydrated in decreasing ethanol concentrations, and stained with hematoxylin and eosin (HE), anti-CD68 (ab125212, abcam, Cambridge, UK), anti-CD45R (RA3-6B2, BD Biosciences, Heidelberg, Germany), anti-CD3 (A 452, DAKO, Glostrup, Denmark), or anti-NE (ab68672, abcam, Cambridge, UK) as described (35). IHC slides were counterstained with hemalaun, and all slides were dehydrated through graded ethanols, cleared in xylene, and coverslipped.

The stained lung slides were digitalized by an Aperio CS2 (Leica Biosystems Imaging Inc., Vista, CA, USA) pathology scanner for subsequent digital image analyses (35). An adapted Aperio GENIE histology pattern recognition algorithm (Leica Biosystems Imaging Inc.) was used for the determination of the affected lung areas, and the Aperio v9 nc algorithm (Leica Biosytems Imaging Inc.) was employed for the quantification of total numbers of nuclei or cells stained by IHC (35).

### Cfu Assay

Untreated WT and PGLYRP4KO PMNs or PMNs pretreated with supernatants (cell- and bacteria-free) from *S. pneumoniae*-stimulated WT and PGLYRP4KO AECs were used for cfu assay. Pneumococci were opsonized by incubation with mouse serum (for WT PMNs with serum from WT mice and for PLYRP4KO PMNs with serum from PGLYRP4KO mice) for 1 h at 37°C and then added to the PMNs. Infection was done with 10^6^ cfu/ml D39 for 1 h. The cells were lysed with Triton X-100 in HBBS Ca^2+^/Mg^2+^, serial dilutions were plated on Columbia agar plates with 5% sheep blood, and incubated overnight (37°C, 5% CO_2_). The next day colonies were counted and cfu's were calculated.

### Microbiota Analysis

#### Sample Preparation

All microbiota analysis experiments were done under a laminar flow bench, and instruments as well as the bench were UV-irradiated for 30 min. The mice were anesthetized i.p. with ketamine and xylazine and euthanized by cutting the *vena cava caudalis* as described above. Samples were put in samples tubes (Biosphere SafeSeal tubes, Sarstedt), snap frozen in liquid nitrogen and stored at−80°C until further processing. After each mouse, instruments were cleaned by rinsing them with distilled water and 70% ethanol. Appropriate control samples were taken the same way.

#### DNA Extraction

Microbial DNA extractions were performed using the DNeasy PowerLyzer PowerSoil Kit (QIAGEN, Hilden, Germany) according to manufacturer's instruction. Briefly, 0.150 g caecum samples were added into the bead tube, 750 μl of bead solution and 60 μl solution C1 were added, mixed, and incubated (10 min, 70°C). A mechanical lysis step was added and done with the FastPrep-24 Homogenizer (MP, 6.5 m/s, 45 s). The purification was done according to the manufacturer's protocol. The DNA was eluted with 100 μl of solution C6, centrifuged (10,000 × g, 1 min), and UV light irradiated (30 min) to minimize foreign DNA. Negative kit, as well as PCR controls were performed using only water and elution buffer (solution C6). In addition, mock communities were run along as a standard and a control for contamination.

#### Library Preparation for Sequencing on MiSeq Illumina Platform

The V4 of the 16S rRNA gene was amplified using primer taken from Caporaso et al. (36). The amplification was done using the Platinum SuperFi PCR Master Mix (Thermo Fisher Scientific, Carlsbad, USA.). Each PCR reaction results in a total of 25 μl reaction solution composed of 2x SuperFi PCR Master Mix, 1.25 μl of 10 pmol forward and reverse primer, and a maximum of 10 μl DNA per reaction. After mixing the solutions, the following thermocycler conditions were run: 2 min at 98°C, 25 cycles with 10 s at 98°C, 10 s at 55°C, and 30 s at 72°C followed by a final 5 min step at 72°C.

After amplification the PCR products were confirmed to be of the right size on a 1% agarose gel. The products were purified using AMPure XP DNA beads (Beckman Coulter, Brea, USA.). The index and adapter ligation PCR was done using the Nextera XT Index Kit V2 Set A and B (Illumina, San Diego, USA) and performed according to the manufacturer's protocol.

The PCR conditions were set as follow: 3 min at 95°C, 8 cycles with 30 s at 95°C, 30 s at 55°C, and 30 s at 72°C plus a final step for 5 min at 72°C. Quality and quantity control of purified PCR products were done using the Qubit instrument (Thermo Fisher Scientific, Waltham, USA) and the 2100 Bioanalyzer instrument (Agilent Technologies, Santa Clara, USA). All samples were diluted to the same molarity (2 nM), pooled, spiked with an internal 15% PhiX control, and paired-end sequenced on the MiSeq Illumina platform using a Nextera V2 cartridge and chemistry with 500 cycles.

#### Bioinformatics Microbiota Workflow

MiSeq Software v3.0 was used to split the sequences by barcode and to generate the fastq files. The microbiota analysis was done following the MiSeq SOP (https://www.mothur.org/wiki/MiSeq_SOP, date accessed: 2018-06-01) using Mothur (version 1.36.1) (37).

The paired end reads were joined, and the primer sequences were removed. We filtered for the expected amplicon length, and removed reads with either ambiguous base calls or with homopolymers longer than eight nucleotides. Duplicate sequences were merged. The unique reads were aligned against the SILVA-bases bacterial reference alignment (38). Nucleotides outside the expected alignment region were trimmed. Reads with a difference of up to two nucleotides were merged during pre-clustering. Chimeric reads were removed using the Mothur implementation of the uchime algorithm. After chimera removal the taxonomy was assigned, and non-bacterial reads were removed. The operational taxonomic units (OTUs) were created using the cluster split method of Mothur. After clustering, we reassigned the taxonomy and removed low abundance OTUs.

Principal coordinates analysis (PCoA) was performed to determine the similarity of samples to each other based on the unweighted unifrac (39) distance. Confidence intervals were calculated using the R package ellipse. Differentially abundant OTUs between groups were computed using linear discriminant analysis (LDA) effect size (LEfSe) (40).

### Data Analysis

For statistical analysis Prism 6 was used (GraphPad Software, San Diego). Samples were categorized by Kolmogorov-Smirnov normality test (threshold at *p* = 0.05) to one of the following distributions: (A) Gaussian, (B) log-normal, (C) other non-Gaussian. Statistical significance (*p* ≤ 0.05) was then analyzed by appropriate tests as stated in the respective figure legends. For microbiota analysis, the linear discriminant analysis effect size (LEfSe) implementation of Mothur was used to compare the OTUs of the different groups.

## Results

### PGLYRP4KO Mice Have an Enhanced Bacterial Clearance

It has been previously published that PGLYRP4 has an antibacterial function against various gram-positive and gram-negative bacteria, and recombinant human PGLYRP4 protected mice from *Staphylococcus aureus-*induced lung infection (11). Interestingly, PGLYRP4KO mice, which were transnasally infected with 10^5^ cfu *S. pneumoniae* per mouse, had a significant decrease in bacterial loads by approximately two-log in the lung (Figure 1A), approximately three-log in the blood (Figure 1B), and a tendency for a small decrease in the spleen (Figure 1C) compared to WT mice 48 h post infection. Furthermore, less PGLYRP4KO mice showed bacteremia (Figure 1D), and they also showed lower clinical scores (Figure 1E) compared to WT mice.

### *Streptococcus pneumoniae*-Infected Lungs of PGLYRP4KO Mice Display Stronger Inflammation and Increased Cell Counts *in vivo*

Histological examination revealed increased tissue damage as displayed by larger areas of perivascular (left and center panel), interstitial and alveolar (right panel) necrosis and hemorrhage accompanied with increased immune cell infiltration, as well as prominent perivascular edema (left and center panel) in infected PGLYRP4KO vs. WT mice (Figure 2A).

A quantitative digital image analysis resulted in significantly more expansive affected/inflamed lung areas (Figure 2B) in infected PGLYRP4KO vs. WT mice with significantly higher total cell counts (Figure 2C).

By histochemical immuno-staining and quantification by digital image analyses we could detect an increase in the number of T cells (Figure 2D, CD3^+^), B cells (Figure 2E, CD45R^+^), and neutrophils (Figure 2F, NE^+^) but not in the monocyte/macrophage population (Figure 2G, CD68^+^) in the lungs of infected PGLYRP4KO mice compared to infected WT mice.

### *Pglyrp4* Expression Is Reduced in Alveolar Epithelial Cells and Induced in Macrophages by *S. pneumoniae*

**Figure 3:**
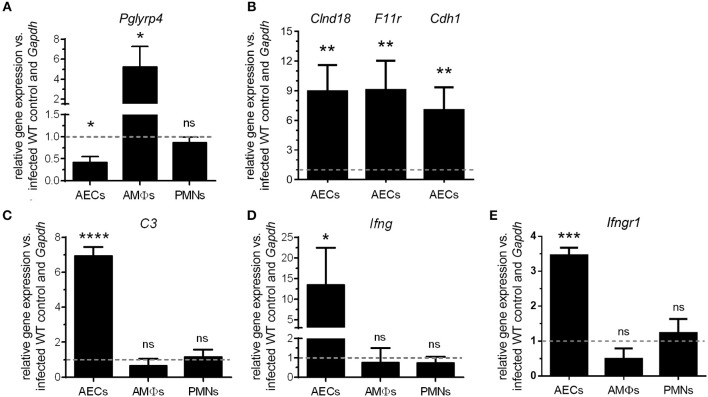
Differential regulation of *Pglyrp4* and candidate gene expression in primary cells. The *Pglyrp4* expression was measured by qPCR in *in vitro*-stimulated (*S. pneumoniae* D39, MOI 1, 6 h) vs. unstimulated WT primary **(A)** alveolar epithelial cells (AECs), alveolar macrophages (AMΦs), and bone marrow-derived neutrophils (PMNs). **(B)** The expression of the tight junction genes *Cldn18, F11r*, and *Cdh1* in PGLYRP4KO vs. WT AECs as well as **(C)** complement *C3*, **(D)** *Ifng*, and **(E)** *Ifngr1* in PGLYRP4KO vs. WT AECs, AMΦs, and PMNs was analyzed after infection (*S. pneumoniae* D39, MOI 1, 6 h). Relative expression was calculated by the ΔΔ*C*_*T*_ method with *Gapdh* as the housekeeping gene and uninfected WT cells as the control. Values are expressed as means + SEMs (*n* = 3–5). The dotted line represents the level of uninfected WT cells. Statistical analysis: Student's *t*-test: ^*^p ≤ 0.05, ^**^p ≤ 0.01, ^***^p ≤ 0.001, ^****^p ≤ 0.0001, ^ns^*p* ≥ 0.1 vs. untreated control.

To investigate possible underlying mechanisms for the lower bacterial burden as well as the higher inflammation and cell recruitment in the lungs of PGLYRP4KO mice, we first analyzed the expression of *Pglyrp4* in resident primary lung cells and recruited innate cell populations. The relative expression of *Pglyrp4* was determined in alveolar epithelial cells (AECs), alveolar macrophages (AMΦs), and bone marrow-derived neutrophils (PMNs). *Pglyrp4* was found to be expressed in all tested cell types (Figure 3A). Upon infection with pneumococci, *Pglyrp4* was significantly downregulated to approximately 40% of the untreated control value in AEC and increased significantly 4-fold in AMΦs (Figure 3A). However, in PMNs the expression of *Pglyrp4* did not change (Figure 3A).

Next, we used global gene expression microarrays to get preliminary data on regulated genes in AMΦs (Figure S1) and AECs (Figures S2–S5) for elucidating a possible mechanism of the improved bacterial clearance and increased lung damage in infected PGLYRP4KO mice compared to WT controls.

The microarray of AMΦs did not reveal any candidate genes (Figure S1). In contrast, the complement component *C3*, the gamma interferon (*Ifng*) (Figure S4) as well as the tight junction genes *Clnd18, F11r*, and *Cdh1* (Figure S5), had higher expression levels in AECs from PGLYRP4KO compared to WT mice. Therefore, these genes were analyzed by qPCR in AECs. All these genes were significantly upregulated in AECs of PGLYRP4KO compared to WT mice (Figures 3B–E). To show the exclusive role of AECs, *C3, Ifng*, and *Ifngr1* were also analyzed in AMΦs and PMNs. No major changes in the expression levels of these genes were detected (Figures 3C–E).

### PGLYRP4-Deficiency Leads to a Pro-inflammatory Cytokine Response

Since we observed more inflamed lung tissue in the histopathological analysis, we were interested if this correlated with higher levels of pro-inflammatory cytokines released by resident and recruited cells in the lung. Therefore, we analyzed the pneumococci-dependent cytokine response in different primary cell types.

**Figure 4:**
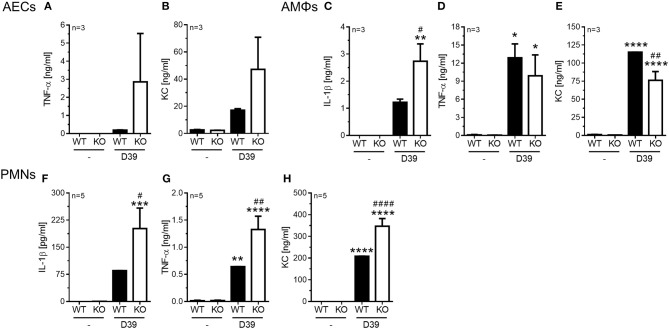
Enhanced pro-inflammatory cytokine release in PGLYRP4KO vs. WT cells. Different cell types were analyzed 6 h after *in vitro*-stimulation with *S. pneumoniae* (D39; MOI 1). In alveolar epithelial cells from either WT or PGLYRP4KO (KO) mice **(A)** TNF-α, **(B)** KC, in alveolar macrophages **(C)** IL-1β, **(D)** TNF-α, and **(E)** KC, as well as in bone morrow-derived neutrophils **(F)** IL-1β, **(G)** TNF-α, and **(H)** KC were analyzed. Values are expressed as means + SEMs (*n* ≥ 3 as indicated in the graphs). Statistical analysis: One-way ANOVA with Holm-Sidak correction for multiple comparisons: ^*^p ≤ 0.05, ^**^p ≤ 0.01, ^***^p ≤ 0.001, ^****^p ≤ 0.0001 vs. untreated control. ^#^*p* ≤ 0.05, ^##^*p* ≤ 0.01, ^####^*p* ≤ 0.0001 vs. infected WT. KO = PGLYRP4KO.

As expected, the stimulation with *S. pneumoniae* induced a strong cytokine response in WT AECs, AMΦs, and PMNs (Figures 4A–H). Interestingly, the pneumococci-dependent cytokine response was altered by PGLYRP4KO in all tested cell types.

In AECs we found a tendency for an increase of TNF-α (Figure 4A) and KC (Figure 4B) in infected PGLYRP4KO compared to infected WT cells, while IL-1β was not detectable (data not shown).

In AMΦs the pneumococci-dependent IL-1β secretion was significantly enhanced (Figure 4C), the TNF-α response was not altered (Figure 4D), and the KC production was significantly reduced (Figure 4E) in infected PGLYRP4KO compared to infected WT cells.

In the bone marrow-derived PMNs significantly greater amounts of IL-1β (Figure 4F), TNF-α (Figure 4G), and KC (Figure 4H) were detectable in the *S. pneumoniae*-stimulated PGLYRP4KO compared to the WT cells.

### Improved Activation of PMNs Caused by Pro-inflammatory Cytokines in PGLYRP4KO AECs

**Figure 5:**
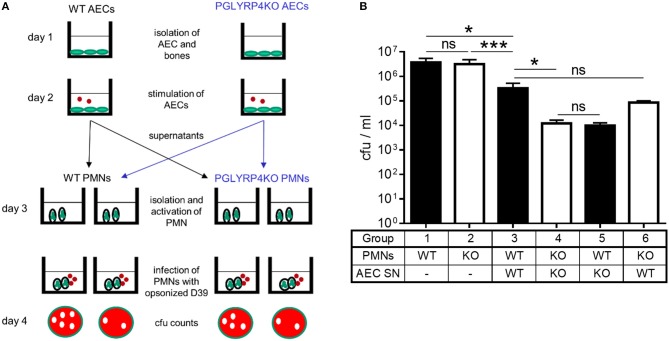
Killing of *S. pneumoniae* by neutrophils is enhanced by supernatants of PGLYRP4KO alveolar epithelial cells. Neutrophils were isolated from the bone marrow and their capacity for killing of *S. pneumoniae* D39 was analyzed in several conditions. **(A)** Scheme of the experimental setting and **(B)** surviving bacteria after incubation with PMNs or activated PMNs (D39, MOI 100, 1 h). First, WT and PGLYRP4KO PMNs were analyzed alone. Second, WT and PGLYRP4KO PMNs were pretreated with respective sterile filtered AEC supernatants, which were infected with *S. pneumoniae* (D39, MOI 1, 16 h). Third, a cross-over experiment was conducted using WT PMNs pretreated with PGLYRP4KO AEC supernatant and PGLYRP4KO PMNs pretreated with WT AEC supernatant. Values are expressed as means + SEMs (*n* = 3 for PMNs alone and *n* = 5-6 for all other experiments). Statistical analysis: One-way ANOVA with Holm-Sidak correction for multiple comparisons on logarithmic data: ^*^p ≤ 0.05, ^***^p < 0.001, ^ns^*p* ≥ 0.1. KO = PGLYRP4KO.

Because there were less bacteria in the lungs and the blood, an increased pro-inflammatory cytokine release by PMNs, and more recruited PMNs detected, as shown by lung histology in PGLYRP4KO vs. WT mice, we hypothesized that the recruited PMNs might be more activated. To corroborate this, WT and PGLYRP4KO PMNs were analyzed *in vitro* for their killing capacity (Figure 5). There was no difference in the efficiency of WT and PGLYRP4KO PMNs to kill *S. pneumoniae* (Figure 5B).

Since AECs are in the first line of defense in the lung, show higher expression of interferon-related genes, *Ifng*, complement factors, and might release more pro-inflammatory cytokines, they could be responsible for a hypothesized higher activation of recruited PMNs. To analyze this, sterile-filtered supernatant from primary AECs infected with *S. pneumoniae* was used to pre-incubate PMNs before the killing assay was performed (Figure 5).

There was an enhanced clearance by WT and PGLYRP4KO PMNs stimulated with the respective AEC supernatant compared to naive cells, whereas the clearance was higher in PGLYRP4KO PMNs which were pre-incubated with the supernatant from PGLYRP4KO AECs compared to WT PMNs (Figure 5B).

To prove that the effect was due to the phenotype of the AECs and not to the genotype of the PMNs, a crossover experiment was done. For this purpose, WT PMNs were pre-incubated with the AEC supernatant from PGLYRP4KO cells and *vice versa*. As expected, there was an enhanced killing in both conditions with a higher clearance in WT PMNs stimulated with PGLYRP4KO AEC supernatant (Figure 5B).

### Analysis of the Caecal Microbiome From WT and PGLYRP4KO Mice

It was previously reported that the gut microbiota is altered in PGLYRP4KO mice, and that some altered bacteria species from this microbiota can affect the outcome of disease in PGLYRP4KO mice (19, 21). Therefore, we examined the caecal microbiota by 16S rRNA gene amplicon-sequencing between *S. pneumoniae*-infected and uninfected mice.

**Figure 6:**
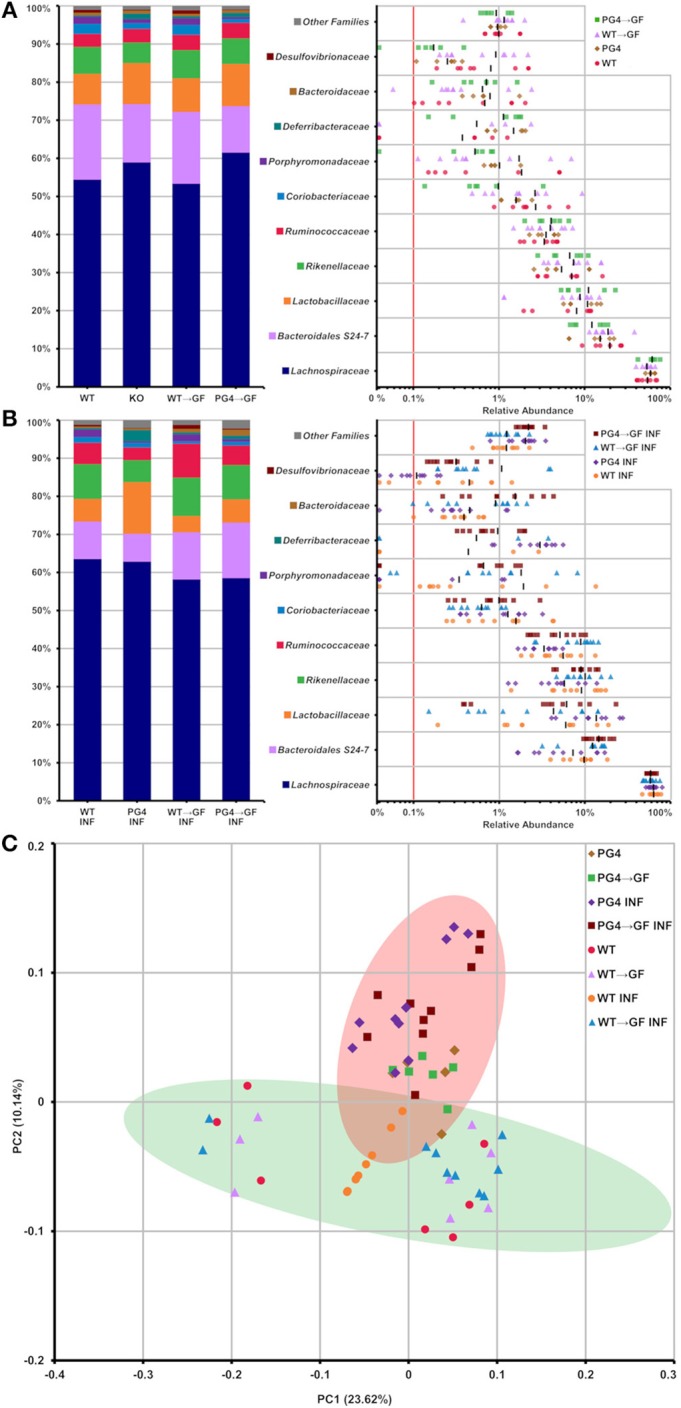
Stable transfer of WT and PGLYRP4KO microbiota into germfree WT mice, and distinct microbiome differences between WT and PGLYRP4KO mice. In **(A,B)** the top 10 families found in caecum from PGLYRP4KO and WT mice are listed. Left: cumulative bar charts comparing relative family abundances. Right: variation in relative abundance of each family. The red line shows cut off for noise. Families not in the top 10 by relative abundance are categorized as other families. **(A)** Uninfected samples and **(B)** samples infected with *S. pneumoniae* (NCTC 7978, 10^5^ cfu, 48 h). **(C)** PCoA plot based on unweighted UniFrac distances for PGLYRP4KO and WT samples infected *S. pneumoniae* and uninfected controls. Green and red circle mark the 95% confidence interval for PGLYRP4KO and WT samples.

Analysis of taxonomic abundance showed well-known gut-related bacteria such as *Lachnospiraceae, Bacteroidales* S24-7, *Lactobacillaceae, Rikenellaceae*, and *Ruminococcaceae* (Figures 6A,B) among the top 10 families. Beta diversity analysis by principal coordinate analysis (PCoA) grouped WT and PGLYRP4KO into two clusters (Figure 6C) which could be significantly distinguished by OTUs using linear discriminant analysis (LDA) score plots (Figures S6, S7).

Next, we were interested if these changes in the microbiota could influence the outcome of pneumococcal lung infection. Therefore, we set up a cohousing experiment to transfer the microbiota from WT mice into germfree WT mice (WT → GF) and from PGLYRP4KO mice into germfree WT mice (KO → GF).

The microbiota transfer from both groups, non-infected and infected, WT into WT → GF mice and from PGLYRP4KO into KO → GF mice resulted in a comparably stable microbiota profile which was indicated by a similar relative abundance pattern of the top 10 families (Figures 6A,B).

### Transfer of the PGLYRP4KO Microbiota Into Germfree WT Mice Enhances Clearance of *S. pneumoniae* in the Lungs and Reduces Clinical Score

We saw a successful and stable transfer of microbiota into the germfree WT mice. Increasing data implicate an influence of gut microbiota on distant organs such as the brain and the lung via SCFA (24, 27, 41). Therefore, we next analyzed the bacterial loads of infected cohoused mice.

**Figure 7:**
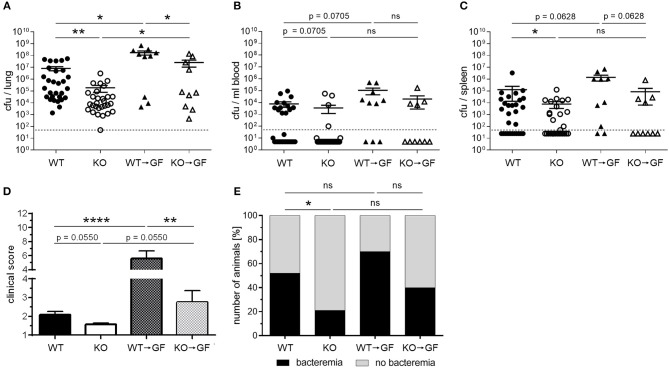
Germfree WT mice with PGLYRP4KO microbiota have an enhanced bacterial clearance in the lungs and lower clinical scores than WT → GF mice. Germfree WT mice were cohoused together with either WT or PGLYRP4KO mice from age 3-4 weeks until an age of 9-10 weeks. Then, all animals were infected with *S. pneumoniae* (NCTC 7978, 10^5^ cfu, 48 h) and **(A)** the lungs, **(B)** the blood, and **(C)** the spleens were analyzed for the bacterial burden. **(D)** The clinical score (min.: 1, max.: 12) and **(E)** the percentage of bacteremic mice were assessed. In **(A–C)** the bars represent the means ± SEMs and in **(D)** the mean + SEM is given. A combined analysis with all animals used in this study was done. In **(A–C)** and **(E)** 29 WT and PGLYRP4KO mice and each 10 WT → GF and KO → GF mice were used. In **(D)** additionally four WT and PGLYRP4KO mice were analyzed. Statistical analysis: **(A–D)** One-way ANOVA with Holm-Sidak correction for multiple comparisons on logarithmic data, and **(E)** Chi^2^-test with number of animals; ^*^p ≤ 0.05, ^**^p ≤ 0.01, ^****^p ≤ 0.0001, ^ns^*p* ≥ 0.1. KO = PGLYRP4KO.

Infection of WT, PGLYRP4KO, and former germfree mice colonized with wildtype (WT → GF), or PGLYRP4KO (KO → GF) microbiota revealed that the WT → GF and KO → GF mice had higher bacterial loads (Figures 7A–C), higher clinical scores (Figure 7D), and slightly higher abundance of bacteremia (Figure 7E) compared to WT and PGLYRP4KO mice.

Comparing WT vs. PGLYRP4KO and WT → GF vs. KO → GF mice, it was evident that KO → GF mice showed the same reduction in bacterial loads in lung (Figure 7A), blood (Figure 7B), and spleen (Figure 7C), clinical score (Figure 7D), and bacteremia (Figure 7E) than PGLYRP4KO mice. However, mean bacterial load in blood between WT and PGLYRP4KO mice shows only a minor difference, which was due to a small proportion of outliers. Median bacterial loads (not shown) and proportion of bacteremia (Figure 7E) is still much lower in PGLYRP4KO vs. WT mice.

## Discussion

We report here an unexpected observation of improved bacterial clearance, hallmarks of pulmonary barrier protection, and enhanced inflammation in mice lacking the host defense factor PGLYRP4. Peptidoglycan recognition proteins have been known about for the last two decades (7, 8). During this time, they were studied extensively in drosophila (9) but also in mammalians including mice and humans (10), and functions, as well as mechanisms of action were characterized in insects and mammalians (9, 10, 42–44).

Mammalian PGLYRP4 was shown to be antibacterial *in vitro* for several gram-positive and gram-negative bacteria including *Bacillus subtilis, Escherichia coli, Lactobacillus acidophilus, Listeria monocytogenes, Proteus vulgaris, Salmonella enterica*, and *Staphylococcus aureus* (11, 12). Because of this broad spectrum of known antibacterial activity, but unknown activity against *S. pneumoniae*, we investigated the *in vivo* effects of PGLYRP4 in a PGLYRP4-deficient mouse pneumonia model.

Interestingly, we observed an approximately 1.5, 3, and 0.5 log lower bacterial burden in the lungs, blood, and spleens of infected PGLYRP4KO compared to WT animals, respectively. This is unexpected in so far as silencing an antibacterial protein should lead to a higher bacterial burden. Therefore, other mechanisms than direct antibacterial functions vs. *S. pneumoniae* must be responsible for the lower bacterial burden. It is known that PGLYRP4 has anti-inflammatory properties (18, 19), and therefore, the PGLYRP4-deficiency could lead to higher inflammation.

Inflammatory processes are essential for the clearance of bacteria by mechanisms such as recruitment and activation of phagocytes (2, 45), but also lead to tissue damage and hyperinflammation (46–48). Patho- and immunohistology revealed higher inflammation in the lungs and more infiltrating cells, including PMNs, T, and B cells.

Higher inflammation might be due to a higher secretion of pro-inflammatory cytokines from PMNs, but AMΦs or AECs could also play a role as seen by our *in vitro* assays. In addition, we showed that PMNs kill more pneumococci, if they are activated by soluble factors from PGLYRP4KO vs. WT AECs. This might be partly due to higher expression of complement (*C3*), *Ifng*, and IFN-regulated and—associated genes, as revealed by microarray and qPCR experiments.

Complement is essential for the elimination of *S. pneumoniae*, and a loss of C3 leads to invasive and recurrent infections (49). However, *S. pneumoniae* uses defense mechanisms against complement. Therefore, an increase might only have minor effects on direct killing (50). IFN-γ, like TNF-α, IL-6, and KC, is a known activator of PMNs (51–53) and therefore, might act synergistically with complement and other soluble factors for the recognition and better clearance of pneumococci by PMNs.

In addition to the enhanced clearance, we showed higher expression of tight junction genes in infected AECs from PGLYRP4KO compared to WT mice. A down-regulation of tight junction proteins is associated with a higher translocation of *S. pneumoniae* through the epithelial barrier (54). Therefore, an induction in tight junction genes indicating a protective effect on pulmonary barrier integrity and might contribute to a reduction of bacteremia in the PGLYRP4KO mice. This might also be modulated partly by IFN-γ, as IFN-γ was shown to have a positive effect on the lung barrier function (55). However, there are only a few reports considering IFN-γ in pneumococcal infection and barrier function in bacterial diseases. Therefore, these results should be taken with care.

Up to this point, it is unclear how PGLYRP4 is modulating the gene expression and pro-inflammatory cytokine release, and if this pro-inflammatory cytokine release is indeed responsible for the better clearance of pneumococci in PGLYRP4KO mice. Recently, several studies revealed that the gastrointestinal microbiota play an important role in the immune response, and the host defense against respiratory diseases underlining the importance of the gut-lung axis related microbiota interactions (25, 56). Schuijt et al. investigated the outcome between gut microbiota-depleted mice and a control group after pulmonary infection with *S. pneumoniae*. In this work, the authors could show that mice with gut microbiota-depletion are more susceptible to infection with *S. pneumoniae*, showing a higher mortality rate and a higher bacterial burden in the lung (25).

Commensal bacteria help to maintain homeostasis of the host, including the immune response. Some commensal bacteria such as *Bacteroidetes* have shown immunomodulatory effects by producing metabolites, e.g., SCFA including acetate, propionate, and butyrate (27).

Those produced SCFA are able to migrate into distant mucosal sites via the blood stream (27), where they can have a systemic influence on distant sites such as the lung to contribute to immunity and health (57).

PGLYRP4KO mice have a lower abundance of *Bacteroidetes* in the gut, resulting in a lower amount of SCFA producing bacteria, which may result in less anti-inflammatory effects. These observations lead to the hypothesis that the PGLYRP4KO mice have a more pro-inflammatory primed immune system caused by the gut microbiota.

Among *Firmicutes*, several *Lactobacilli* species are known to be probiotic and show protective behavior during diseases like urogenital infection caused by *Staphylococcus aureus* (58) and *Salmonella enteritidis* infection (59). In addition to protective effects, *Lactobacilli* species also possess immunomodulatory activities (60). A study investigated the immunomodulatory effects of two *Lactobacillus rhamnosus* strains during infection with *S. pneumoniae* (61). Only one strain was associated with an increase in Th1 and Th2 cytokines, and a decrease of bacterial burden when examining BAL samples, indicating the immunomodulatory effects are *Lactobacillus rhamnosus* strain specific.

In addition, different *Lactobacilli* species (e.g., *Lactobacillus reuteri* and *Lactobacillus crispatus*) of the gastrointestinal tract revealed their ability to promote resistance to lung infection through NOD2 signaling and GM-CSF release (62). Our microbiota analysis indicates that *Lactobacillus* (OTU0002 and OTU00029) is significantly more abundant in the gut of infected PGLYRP4KO compared to WT mice, which might explain the pro-inflammatory phenotype of PGLYRP4KO mice.

Furthermore, we could illustrate that the transfer of the PGLYRP4KO microbiota into germfree WT mice (KO → GF) is sufficient to improve pneumococcal clearance, and significantly reduces the clinical disease score. However, there are discrepancies between the original experiments with WT vs. PGLYRP4KO mice and the cohousing experiments. Mean blood cfu's, and the corresponding statistical analysis, are highly influenced due to outliers in the PGLYRP4KO group. However, the overall interpretation does not change because the PGLYRP4KO mice still show less bacteremia compared to the WT mice.

We therefore conclude that intestinal microbiota shapes and influences the lung immunity. This might be via SCFA release and microaspirated bacteria from the gut which finally contributes to lung immunity to support better pathogen clearance in PGLYRP4KO mice. However, microbiotal changes have to be carefully considered in the disease setting, because we have seen a more pro-inflammatory gut microbiome and a more pro-inflammatory lung milieu. This might be disadvantageous in some diseases like COPD or Crohn's disease but beneficial in other settings like *S. pneumoniae* lung infection.

Further studies will be needed to establish the precise mode of action in which PGLYRP4 shapes the microbiota, how the microbiota mediates its effects and to which extent these effects can be transferred into germ-free mice.

Taken together, our results indicate that lung epithelial cells not only function as barriers and sensors of invading bacteria, but also are capable of activating professional phagocytes for pathogen killing. Furthermore, the microbiota are highly capable of modulating the immune system. Through a loss of PGLYRP4, the (gut) microbiota is altered and this in turn influences gene expressions, pro-inflammatory cytokine release, and immune functions in the lungs to help combat *S. pneumoniae* infections.

## Data Availability

The datasets generated for this study can be found in NCBI's Gene Expression Omnibus (63), GSE108358 (https://www.ncbi.nlm.nih.gov/geo/query/acc.cgi?acc=GSE108358), GSE126065 (https://www.ncbi.nlm.nih.gov/geo/query/acc.cgi?acc=GSE126065), and at NCBI's Sequence Read Archive, PRJNA495123 (https://www.ncbi.nlm.nih.gov/bioproject/PRJNA495123/).

## Ethics Statement

All animal procedures were in compliance with the Federation of European Laboratory Animal Science Associations (FELASA) guidelines and recommendations for the care and use of laboratory animals, and were approved by local institutional (Charité – Universitätsmedizin Berlin) and governmental (LAGeSo Berlin, approval ID: G0251/12 and G0266/15) authorities.

## Author Contributions

PN'G, SA, TH, and JZ: conceptualization. AD, AS, KK, MW, KD, WB, JW, JZ, AG, and TH: formal analysis. SA, JZ, NS, TH, BS, and AG: funding acquisition. AD, AS, CC, and JZ: investigation. KK, MW, and TH: methodology. TH, JZ, and NS: project administration. NS, TH, and BS: resources. PN'G, SA, JZ, KR, and TH: supervision. AD, KK, MW, WB, JW, and KD: visualization. AD, AS, JZ, KK, TH, and MW: writing—original draft preparation. AD, AS, CC, KK, MW, KD, AG, WB, JW, BS, KR, PN'G, SA, NS, JZ, and TH: writing—review and editing.

### Conflict of Interest Statement

The authors declare that the research was conducted in the absence of any commercial or financial relationships that could be construed as a potential conflict of interest.
