# Supplementary material for: Peptidoglycan Recognition Protein 4 Limits Bacterial Clearance and Inflammation in Lungs by Control of the Gut Microbiota
Source: Front Immunol. 2019 Sep 20;10:2106. doi: 10.3389/fimmu.2019.02106 (PMC6763742; doi:10.3389/fimmu.2019.02106)
Supplement: Supplementary file 1 [file Data_Sheet_1.docx]

*Supplementary Material*

**Peptidoglycan Recognition Protein 4 Limits Bacterial Clearance and Inflammation in Lungs by Control of the Gut Microbiota**

Alexander N. Dabrowski, Anshu Shrivastav, Claudia Conrad, Kassandra Komma, Markus Weigel, Kristina Dietert, Achim D. Gruber, Wilhelm Bertrams, Jochen Wilhelm, Bernd Schmeck, Katrin Reppe, Philippe Dje N’Guessan, Sahar Aly, Norbert Suttorp, Torsten Hain, and Janine Zahlten*

*** Correspondence:** Janine Zahlten: Janine.Zahlten@charite.de

# Supplementary results

## Loss of PGLYRP4 has an impact on the regulation of IFN-signaling related molecules in alveolar epithelial cells

We were interested in whether there were changes in the global gene expression profile of alveolar macrophages (AMΦs) and alveolar epithelial cells (AEC), because i) in these cells *Pglyrp4* expression was significantly altered upon infection with *S. pneumoniae* (**Figure 3A**), ii) they are the first line of defense against pathogens invading the lung, and iii) AECs are one of the major population of cells in the lungs. Therefore, uninfected and *S. pneumoniae-*stimulated WT and PGLYRP4KO AMΦs and AECs were analyzed using a mouse gene expression microarray. Analyses of the AMΦs revealed only 9 and 19 up- and downregulated genes in uninfected PGLYRP4KO vs. WT **(Supplementary Figure 1A)** as well as 9 and 6 annotated up- and downregulated genes in infected PGLYRP4KO vs. WT (**Supplementary Figure 1B**) in the top 50 regulated genes. Within these genes none could sufficiently explain the observed phenotype. The analysis of AECs revealed that, beside other pathways, cell adhesion molecules and the ‘Herpes simplex infection’ pathway were mostly affected in the *S. pneumoniae*-stimulated PGLYRP4KO compared to WT AECs (**Supplementary Figure 2**). Furthermore, some interferon-related or interferon-regulated genes were upregulated in uninfected as well as infected PGLYRP4KO cells compared to WT cells (**Supplementary Figure 3**). These genes include *Gbp5*, *Ifit1*, *Ifnb1, Mx2, Pyhin1*, and *Themis2* in uninfected cells, *Batf2, Isg15, Ly6c1*, and *Slfn5* in infected cells, and *Cxcl10*, *Fcgr1*, *Ifi205*, *Ifi44*, *Ifi44l*, *Ifit2*, *Ifit3*, *IIgp1*, *Oasl1*, *Rsad2*, and *Slfn4*, in both, uninfected and infected cells. In the ‘Herpes simplex infection’ pathway there was, e.g., complement *C3* and *Hc*, as well as *Ifng* and *Ifna4* upregulated in PGLYRP4KO compared to WT cells (**Supplementary Figure 4**). Pathway maps of the cell adhesion molecules demonstrated that tight junction proteins were mostly upregulated in PGLYRP4KO compared to WT cells (**Supplementary Figure 5**).

# Supplementary figures


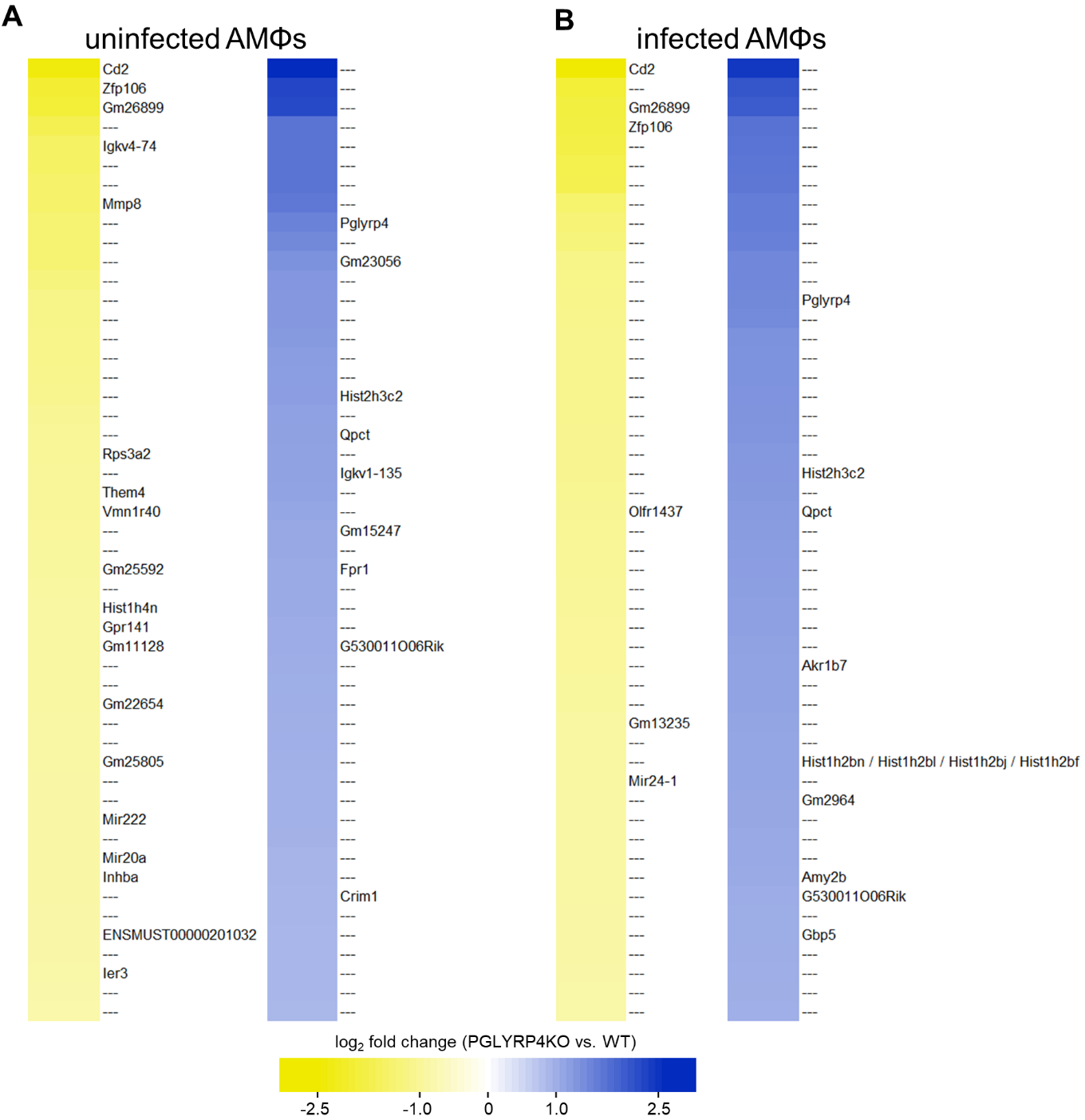


Figure S1**: Top 50 up- and downregulated genes by microarray analysis of WT and PGLYRP4KO alveolar macrophages.**

**A)** Unstimulated and **B)** *S. pneumoniae*-stimulated (D39; MOI 1, 6 h) AMΦs were analyzed for whole genome RNA transcription. The top 50 up- and downregulated genes are ranked by log_2_ fold change after Student’s t test (p ≤ 0.05). Two samples for uninfected WT and three samples for all other conditions. Genes which could not be annotated are indicated with a dashed line (‑‑‑).


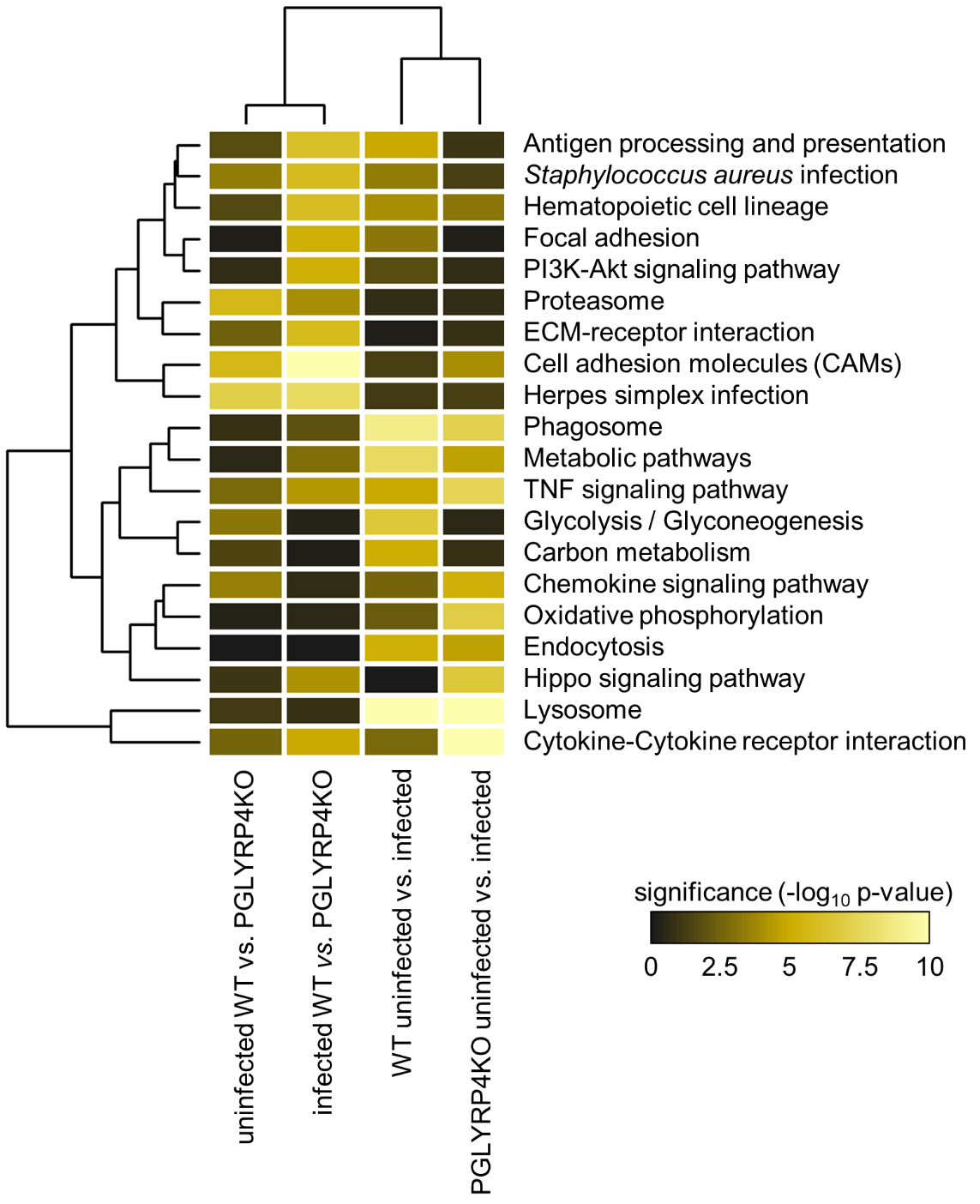


Figure S2: **Hierarchic pathway clustering of gene expression in WT vs. PGLYRP4KO alveolar epithelial cells.**

After stimulated with or without *S. pneumoniae* (D39; MOI 1, 6 h) an RNA microarray was used to analyze whole genome gene expression data. Afterwards, hierarchic pathway clustering of genes was done, and significance was calculated by numbers of differentially expressed genes within a pathway. A pooled analysis of n = 3 was performed. Significance of pathway enrichment is displayed as negative log_10_ p-value.


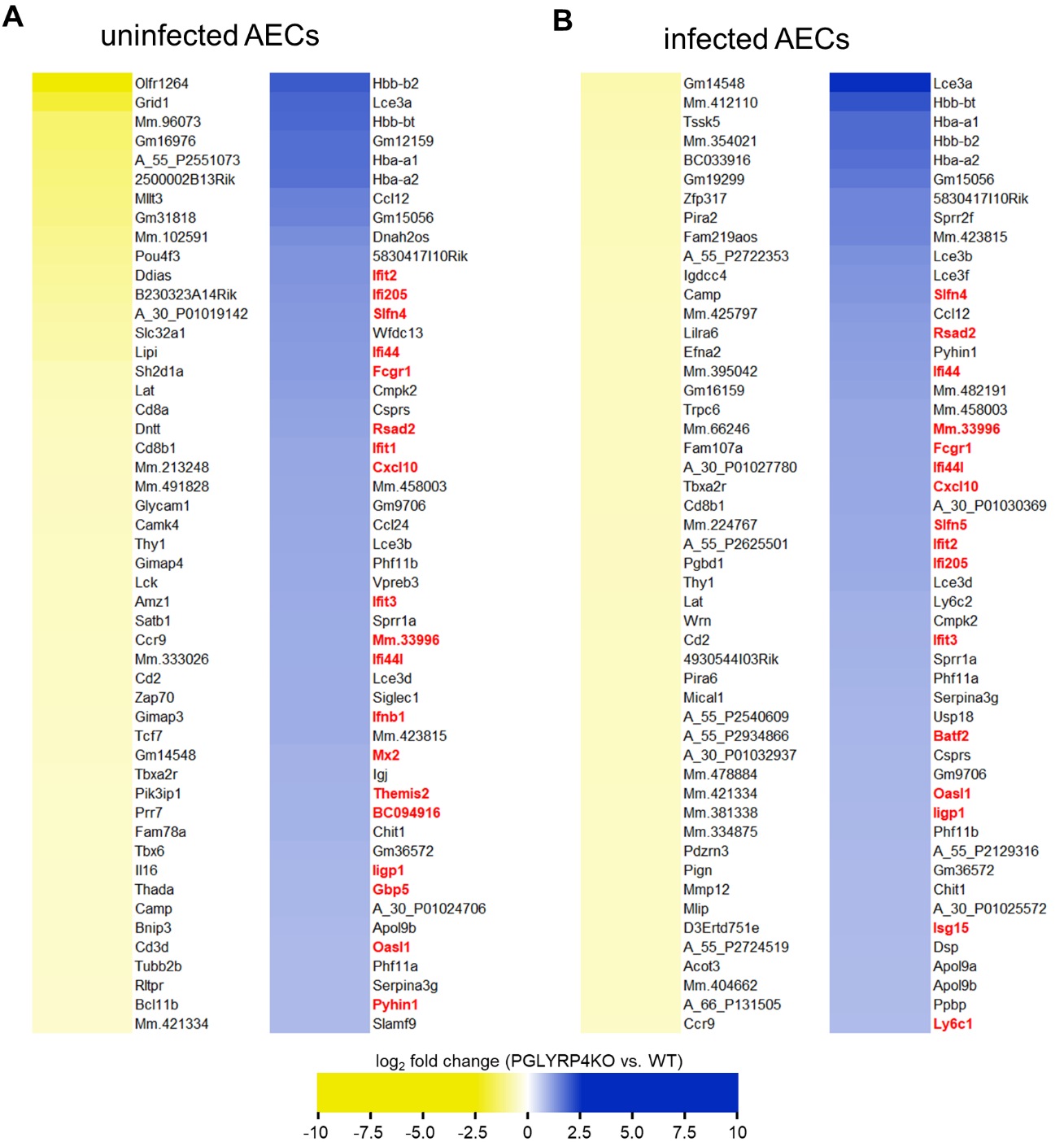


Figure S3**: Top 50 up- and downregulated genes by microarray analysis of WT and PGLYRP4KO alveolar epithelial cells.**

**A)** Uninfected and **B)** infected (*S. pneumoniae* D39; MOI 1, 6 h) AECs were analyzed for whole genome gene expression. The top 50 up- and downregulated genes are ranked by log_2_ fold change. A pooled analysis of n = 3 was performed. Interferon-related genes are typed in bold, red letters.


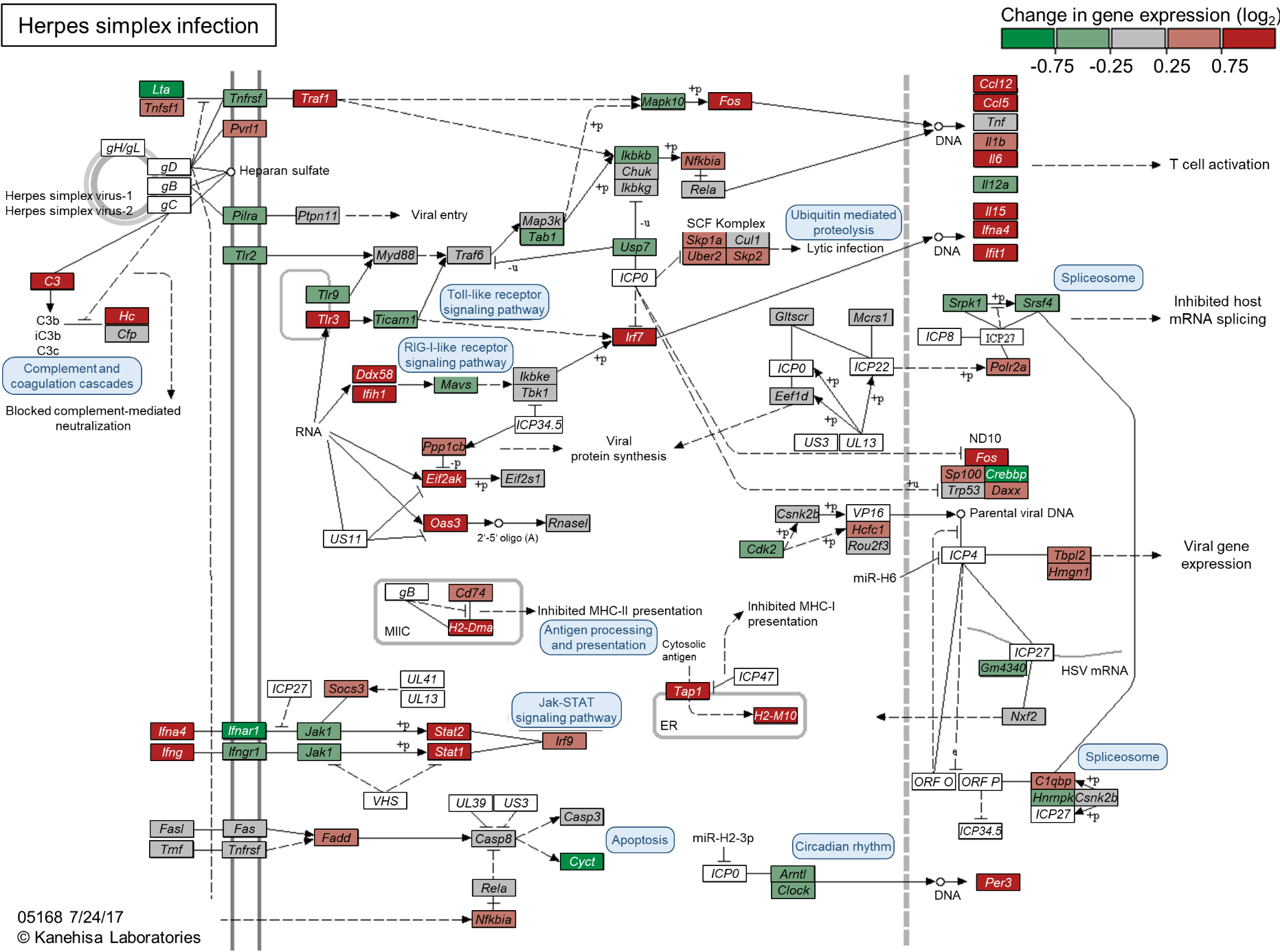
Figure S4: **Gene array data of the ‘Herpes simplex infection’ pathway.**

Alveolar epithelial cells infected with *S. pneumoniae* (D39, MOI 1, 6 h) were analyzed by a whole genome gene microarray. The KEGG pathway map ‘Herpes simplex infection’ (map05168) was used for visualization (1). A pooled analysis of n = 3 was performed. Fold change is indicated as following: Red: upregulated in PGLYRP4KO vs. WT, Green: downregulated in PGLYRP4KO vs. WT.


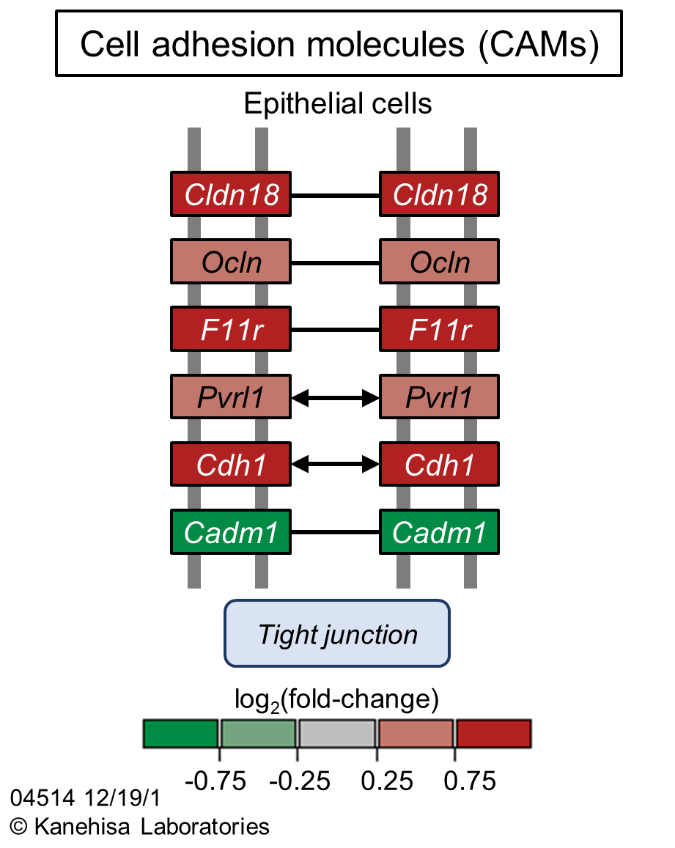


Figure S5**: Gene array data for the epithelial cell part of the ‘cell adhesion molecules pathway’.**

Alveolar epithelial cells infected with *S. pneumoniae* (D39, MOI 1, 6 h) were analyzed by a whole genome gene microarray. The KEGG pathway map ‘Cell adhesion molecules’ (map04514) was used for visualization (1). A pooled analysis of n = 3 was performed. Fold change is indicated as following: Red: upregulated in PGLYRP4KO vs. WT, Green: downregulated in PGLYRP4KO vs. WT.


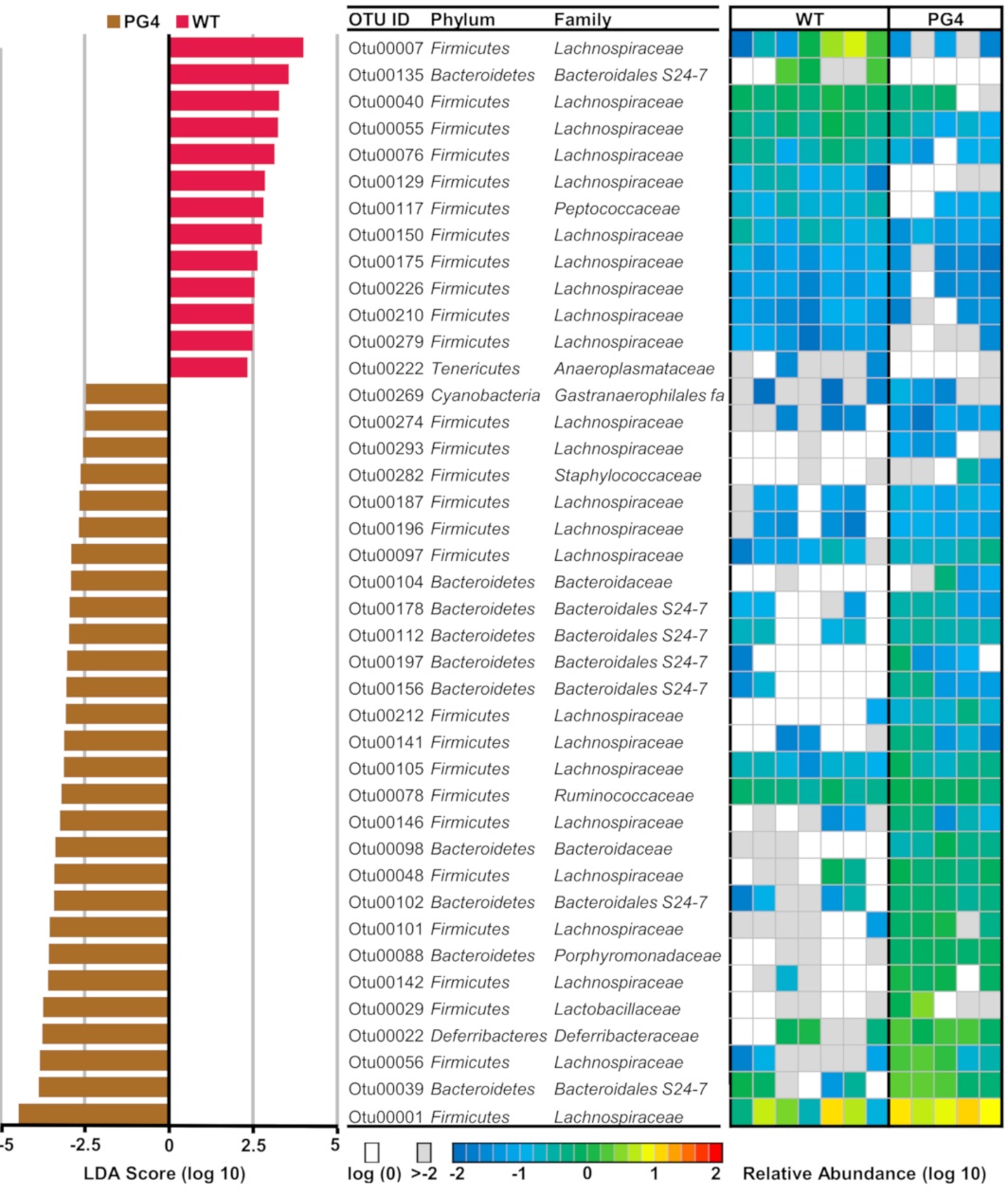


**Figure S6: Distinct microbiome differences between uninfected WT and PGLYRP4KO mice**

The LDA score for significant (p ≤ 0.05) differentially abundant OTUs calculated with linear discriminant analysis effect size (left), the taxonomic classification (center), and a heat map showing the log10 transformed relative abundance for each OTU in the different samples (right) are shown. Comparing uninfected PGLYRP4KO vs. WT mice. KO = PGLYRP4KO.


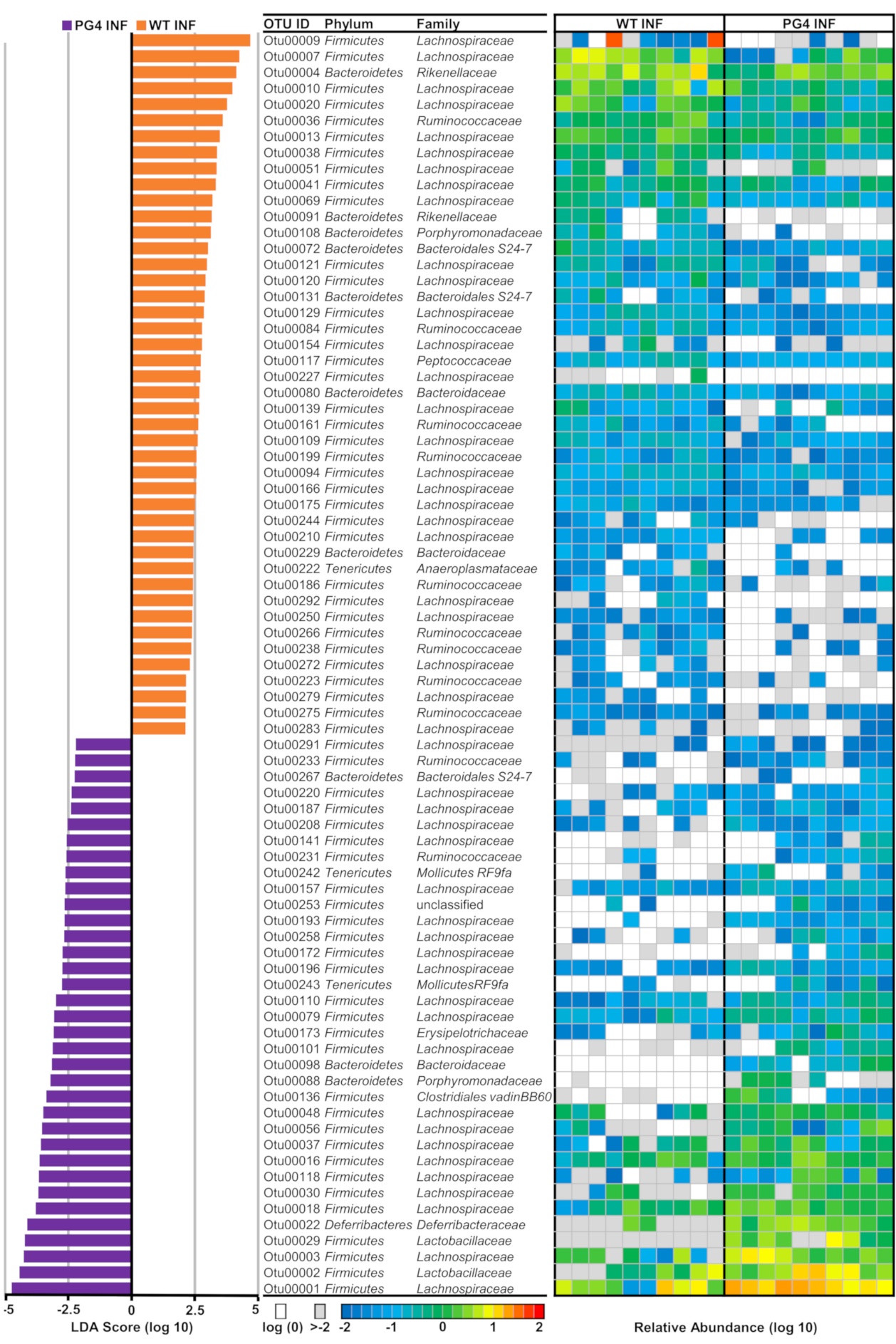


**Figure S7: Distinct microbiome differences between infected WT and PGLYRP4KO mice**

The LDA score for significant (p ≤ 0.05) differentially abundant OTUs calculated with linear discriminant analysis effect size (left), the taxonomic classification (center), and a heat map showing the log10 transformed relative abundance for each OTU in the different samples (right) are shown. Comparing with *S. pneumoniae* (NCTC 7978, 10^5^ cfu, 48 h) infected PGLYRP4KO vs. WT mice. KO = PGLYRP4KO, INF = infected with *S. pneumoniae*.

# Supplemental references

1. Kanehisa M, Goto S. KEGG: Kyoto Encyclopedia of Genes and Genomes. *Nucleic Acids Res* (2000) **28**:27–30. doi:10.1093/nar/28.1.27
